# Assessing anthropogenic heat flux of public cloud data centers: current and future trends

**DOI:** 10.7717/peerj-cs.478

**Published:** 2021-05-05

**Authors:** Hamza Baniata, Sami Mahmood, Attila Kertesz

**Affiliations:** 1 Software Engineering Department, University of Szeged, Szeged, Hungary; 2 Physics Department, The University of Jordan, Amman, Jordan; 3 Department of Physics and Astronomy, Michigan State University, East Lansing, MI, United States of America

**Keywords:** Global warming, Anthropogenic heat flux, Cloud computing, Data centers

## Abstract

Global average temperature had been significantly increasing during the past century, mainly due to the growing rates of greenhouse gas (GHG) emissions, leading to a global warming problem. Many research works indicated other causes of this problem, such as the anthropogenic heat flux (AHF). Cloud computing (CC) data centers (DCs), for example, perform massive computational tasks for end users, leading to emit huge amounts of waste heat towards the surrounding (local) atmosphere in the form of AHF. Out of the total power consumption of a public cloud DC, nearly 10% is wasted in the form of heat. In this paper, we quantitatively and qualitatively analyze the current state of AHF emissions of the top three cloud service providers (i.e., Google, Azure and Amazon) according to their average energy consumption and the global distribution of their DCs. In this study, we found that Microsoft Azure DCs emit the highest amounts of AHF, followed by Amazon and Google, respectively. We also found that Europe is the most negatively affected by AHF of public DCs, due to its small area relative to other continents and the large number of cloud DCs within. Accordingly, we present mean estimations of continental AHF density per square meter. Following our results, we found that the top three clouds (with waste heat at a rate of 1,720.512 MW) contribute an average of more than 2.8% out of averaged continental AHF emissions. Using this percentage, we provide future trends estimations of AHF densities in the period [2020–2100]. In one of the presented scenarios, our estimations predict that by 2100, AHF of public clouds DCs will reach 0.01 Wm^−2^.

## Introduction

Cloud computing has been around for a few decades now, yet it has grown dramatically in the last ten years. This is due to the significant enhancement of applications, hardware, and software technologies. The large number of services provided to end-users, and the reliability and security offered by cloud providers encouraged many companies, organisations, and industries to migrate their services and data to the cloud. Clouds can furnish all needed platforms, software, and infrastructures for users at ease, and relatively low costs.

*Climate* is the average weather, or the statistical description in terms of the mean and variability of its relevant quantities over a period of time ranging from 30 years to thousands or millions of years (IPCC, 2013). *Global Climate Change*, on the other hand, is the change in the state of the climate that can be identified by changes in the mean and/or the variability of its properties (IPCC, 2013). One of the problems that is attracting a global attention is the temperature of Earth’s surface, oceans and atmosphere going up over tens of years, namely *Global Warming*.

The continuous burn of fossils and fuels was found a leading contributor to increased levels in emission of greenhouse gas (GHG), forming a barrier in the face of heat waves emitted towards the atmosphere and resulting in a type of heat storage (Guemas et al., 2013). Heat emitted towards the atmosphere is mainly originated by both solar energy radiated back to the atmosphere (Guermoui et al., 2020), and industrial waste heat from Earth surface due to anthropogenic activities (Letcher, 2019; Jin et al., 2019). Industrial waste heat was defined in Oluleye et al. (2016) as the sum of the residual heat rejected from the processes on a site and residual heat rejected from the site utility system designed to satisfy the energy demand. In other words, when a process and a site have reached their maximum potential for heat recovery, the resulting heat is the waste heat. Waste heat is also named surplus heat or excess heat (Miró, Gasia & Cabeza, 2016), which eventually becomes a part of the heat flux thrown into the close-to-surface layer of the atmosphere. This heat storage phenomenon is one factor, among many, imposing the Global Warming problem, in its big picture, and consequently higher average temperatures than thermal comfort indexes (Zhou et al., 2013). Specifically, it was reported in Jin, Wang & Wang (2020) that the land surface temperature (LST) may increase by 0.0275 °C for each 1.0 W/m^2^ increase in AHF in the context of urbanization (although such increment is conditioned with many other circumstances and was based on eastern China only and may not be the case for every country/continent). The increment in the average temperature was argued by many researchers to be the cause of droughts, heavy rainfalls/flooding around the globe (Garreaud et al., 2020), and named diseases (Duignan, Stephens & Robb, 2020).

**Figure 1:**
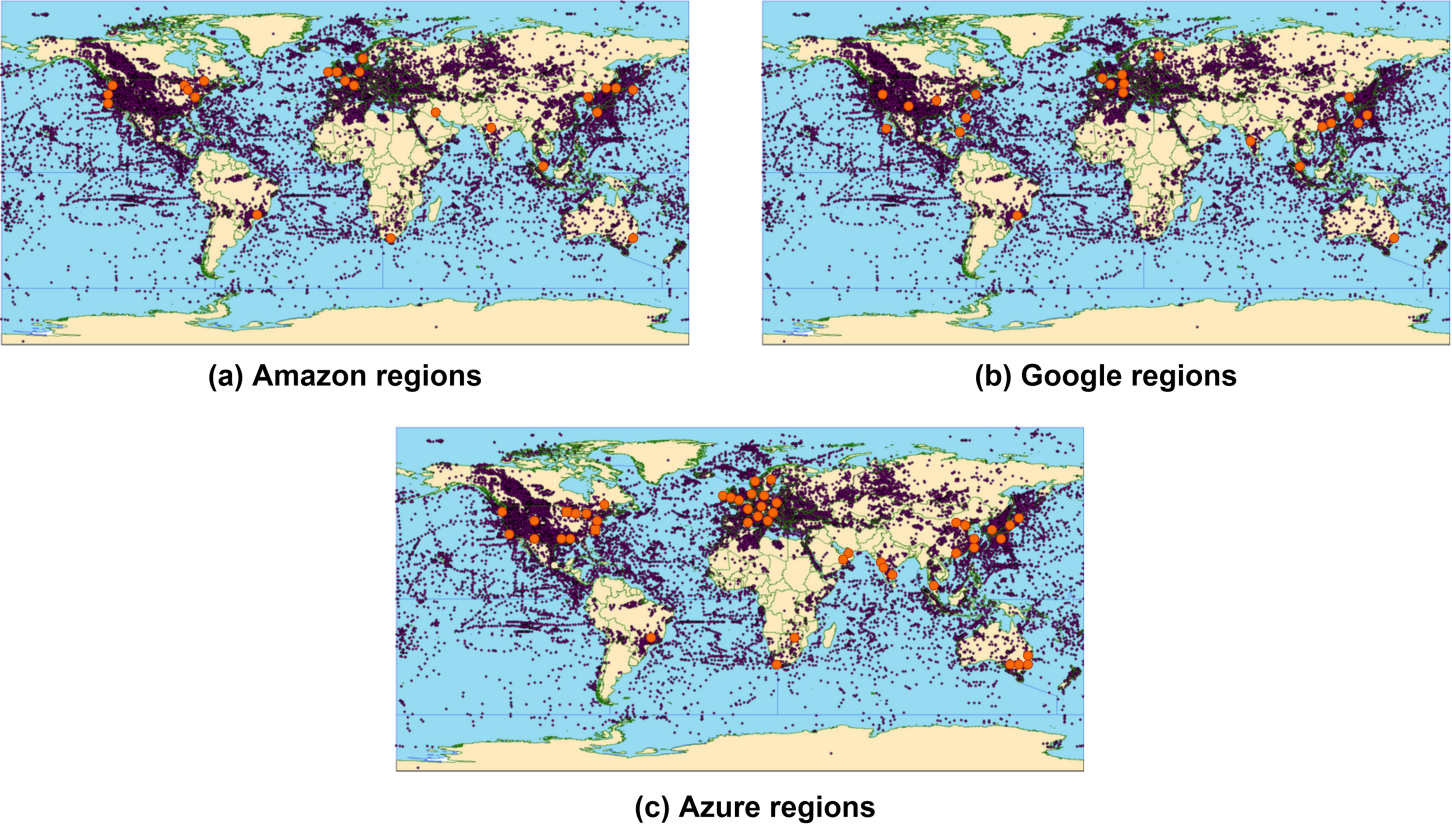
Public clouds regions projected on a global heat flow map. (A) Amazon regions, (B) Google regions (C) Azure regions.

Figure 1 projects the region placement maps of Amazon (https://aws.amazon.com/about-aws/global-infrastructure/), Microsoft Azure (https://azure.microsoft.com/en-us/global-infrastructure/regions/#services) and Google (https://cloud.google.com/about/locations) on a map that presents the current state of global heat flux (http://www.datapages.com/gis-map-publishing-program/gis-open-files/global-framework/global-heat-flow-database). As can be noted, the majority of public cloud service providers’ (CSP) data centers (DCs) are globally located at the most heat emitting regions.^1^ 1Maps were constructed depending on data obtained from the official site of each CSP, last accessed: 19-April-2020.This projection motivated us to analyze the heat flux emitted by top CSPs’ DCs. In this study, we assess the current and future state of waste heat emitted by public clouds DCs. To accurately address our goals, we discuss the general architecture of public clouds, investigate previous works that proposed public clouds DCs energy consumption estimations, and adopt the most relevant estimation according to recent reports on global energy consumption and market shares. Consequently, we deploy recently published clouds efficiency measurements to deduce the wasted fraction of consumed energy, and provide averaged and detailed, global and continental AHF estimations. We then use the detailed continental AHF emissions to assess its effect, and analyze the ratio of averaged global AHF emissions of public clouds, to previously reported average global AHF values. Accordingly, we further present future AHF estimations, emitted by different clouds DCs.

The remainder of our paper is organized as follows: ‘Background’ presents previous related works regarding global AHF estimations and solutions. Additionally, it briefly discusses the architecture of public clouds, DC power consumption, distribution, and efficiency. ‘Methods’ presents the methods and adopted values that we use to asses AHF of public clouds. ‘Results’ presents our assessment and discusses current and future trends of public clouds AHF. Finally, ‘Conclusion’ concludes our work.

## Background

In a typical DC architecture, a fraction of the consumed energy is used for purposes other than performing the computational tasks requested by cloud clients. Examples of such include cooling and networking, which creates a stream of waste heat (Ebrahimi, Jones & Fleischer, 2014) or what is called Anthropogenic Heat Flux (AHF). In this section, we briefly review the state-of-the-art regarding the architecture of public clouds, the power consumption estimations and the distribution of this power in public clouds DCs. Additionally, we discuss how the efficiency of DCs can be analyzed according to measured power consumption and designed power distribution. Furthermore, we discuss previous studies that present global AHF estimations and possible solutions.

According to facts that we adopt by the end of this section, we systematically analyze the AHF specifically contributed by public clouds DCs, and present our predictions regarding future AHF contributions up until the year 2100.

### Architecture of public clouds

**Figure 2:**
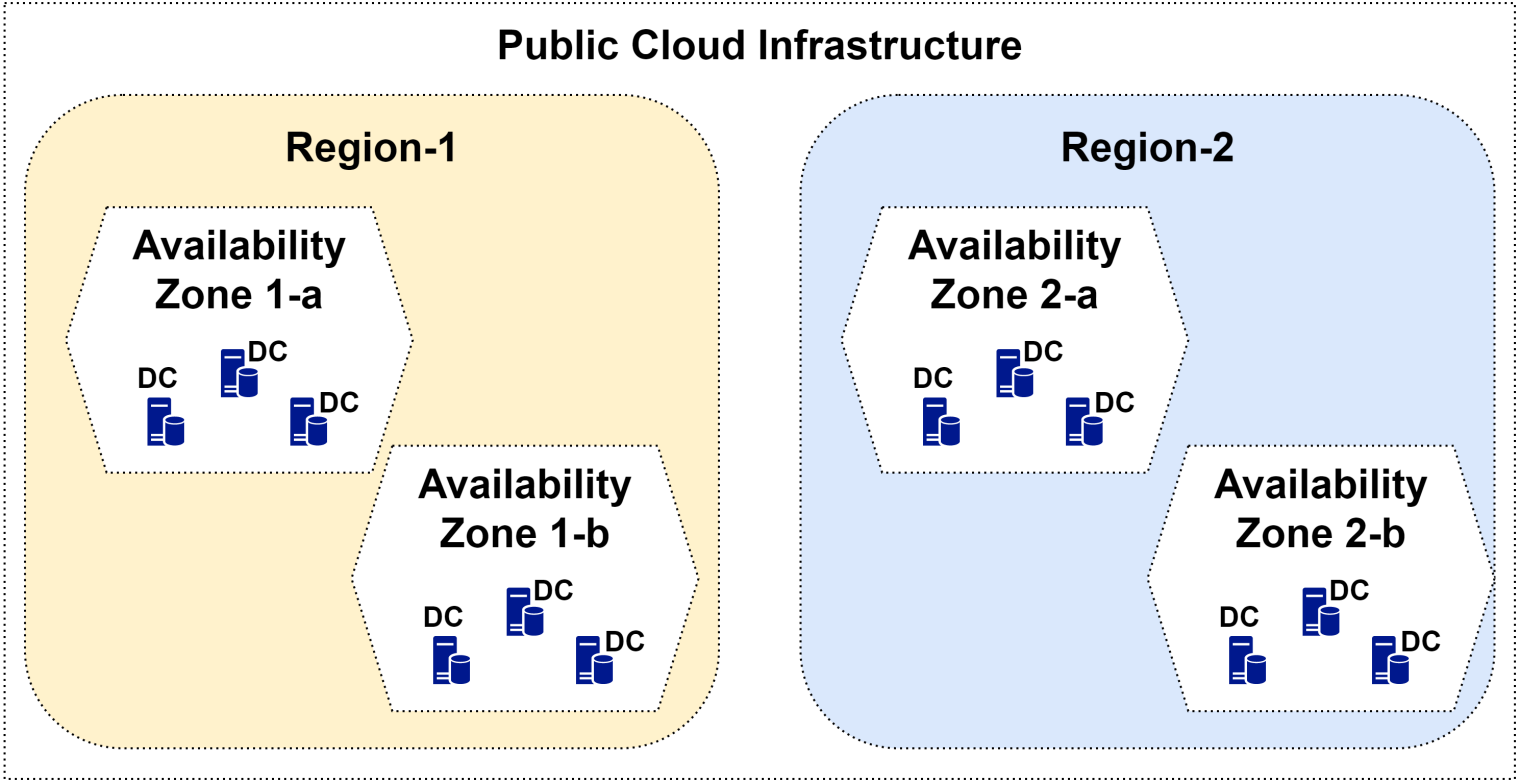
Illustration of cloud infrastructure, including the concepts of regions, availability zones, and data centers.

Major cloud service providers (CSP) (i.e., Google, Microsoft Azure and Amazon) build their infrastructure in a way that would make their services reachable from almost any place on Earth, as long as there is an internet connection. That is, Data centers (DCs) are distributed among distinct regions, which are located in different cities, countries, and continents. Currently,^2^ 2Last checked: 18-April-2020DCs of public CSPs are distributed among 65 locations around the world. As clarified in Fig. 2, each region consists of at least one availability zone, and each zone consists of at least one DC, with its independent energy sources, cooling systems, and communications. Such formation allows virtual networking and increases the availability to reach 99.99% in some cases (Uzaman et al., 2019). Table 1 presents the number of regions and zones currently available from each of the top three CSPs. As it can be noted in the table, the total number of regions is 106, with average number of zones per region ≈ 3. Taking the average number of DCs per zone ≈ 3 (as publicly published on the considered CSPs websites), the total number of DCs that belong to these CSPs ≈ 933 DCs.

### Power consumption of public cloud data centers

Reports indicated that cloud DCs are responsible for more than 2% of the US total electricity usage, and 1–1.5% of global electricity usage (Andrae & Edler, 2015). However, different studies revealed large variations of the energy consumption of the DC industry as illustrated by the results of five different studies (Table 2). The average value of the results of these studies is 208.4 billion kWh/year, with a standard deviation of 116.5 billion kWh/year. Even though this average value is derived from estimates of annual global energy consumption in different periods of time (2010–2020), during which the total number of DCs and the DC technology have changed, it is very close to the result (205 billion kWh/year) recently reported by Masanet et al. (2020) for the global energy consumption of DCs in the year 2018. Additionally, if the projected global DC energy consumption in the year 2020 is estimated from the results provided by Ascierto et al. (2015) and Andrae & Edler (2015), the value of (270 ± 130) billion kWh/year is also consistent with the above mentioned average. The large variations of the reported results in the individual studies is attributed to adopting different numbers of DCs, and different methodologies.

As our intention in this article is to determine the AHF produced by the DCs of the three public CSPs (a total of 933 DCs), knowledge of the estimated energy consumption and efficiency of a single DC is required. Analysis reported by Kaplan, Forrest & Kindler (2008) estimated that a public cloud DC consumes as much energy as 25,000 households. Bearing in mind that the average monthly energy consumption of a household at the time of that study was 339 kWh (Firth et al., 2008), the average energy consumption of one public DC is then estimated by 101.7 × 10^6^ kWh/year. Therefore, the total energy consumption of DCs belonging to the three CSPs is 101.7 ×10^6^ × 933 = 94.9 billion kWh/year. This accounts for 46% of the global average of 208.4 billion kWh/year. Recent reports estimated the global market share^3^ 3As of October 2020: https://www.canalys.com/newsroom/worldwide-cloud-market-q320.of Amazon, Microsoft Azure, and google as 32%, 19%, and 7% of the global CSPs market, respectively. According to these market shares, the three CSPs consume 58% of the global average energy consumption (120.9 billion kWh/year). Consequently, our best estimate of the uncertainty in Kaplan’s estimate of the energy consumption of a single DC is obtained from the difference between these two results ([120.9–94.9]/94.9) × 100% = 27%). According to Kaplan’s estimates, the average power consumption of a public DC ≈ 11.6 MW (± 3.132 MW, bearing in mind that a year is about 8760 h). In fact, this average is in good agreement with the average power consumption of 10.17 MW per DC, during the year 2018, derived from Google’s report Google (2019). In the forthcoming analysis, we will adopt Kaplan’s estimates of the energy consumption by a single DC.

**Table 1:** Reported number of regions, zones, and DCs of top cloud providers.

**Cloud Provider**	**Regions**	**Zones**	**DCs/Zone**	**Total no. DCs**
Google	23	67		201
Azure	57	174	3	522
Amazon	26	70		210
**Total**	**106**	**311**	–	**933**

**Table 2:** Global energy consumption estimations of the DC industry, as provided by previous works.

**Reference**	**Year**	**Estimated Energy Consumption** (kWh/year)
Gao et al. (2012)	2010	201.8 billion
Ascierto et al. (2015)	2015	95 billion
Masanet et al. (2020)	2018	205 billion
Ascierto et al. (2015)	2020	140 billion
Andrae & Edler (2015)	2020	400 billion

### Power distribution and efficiency in public data centers

**Figure 3:**
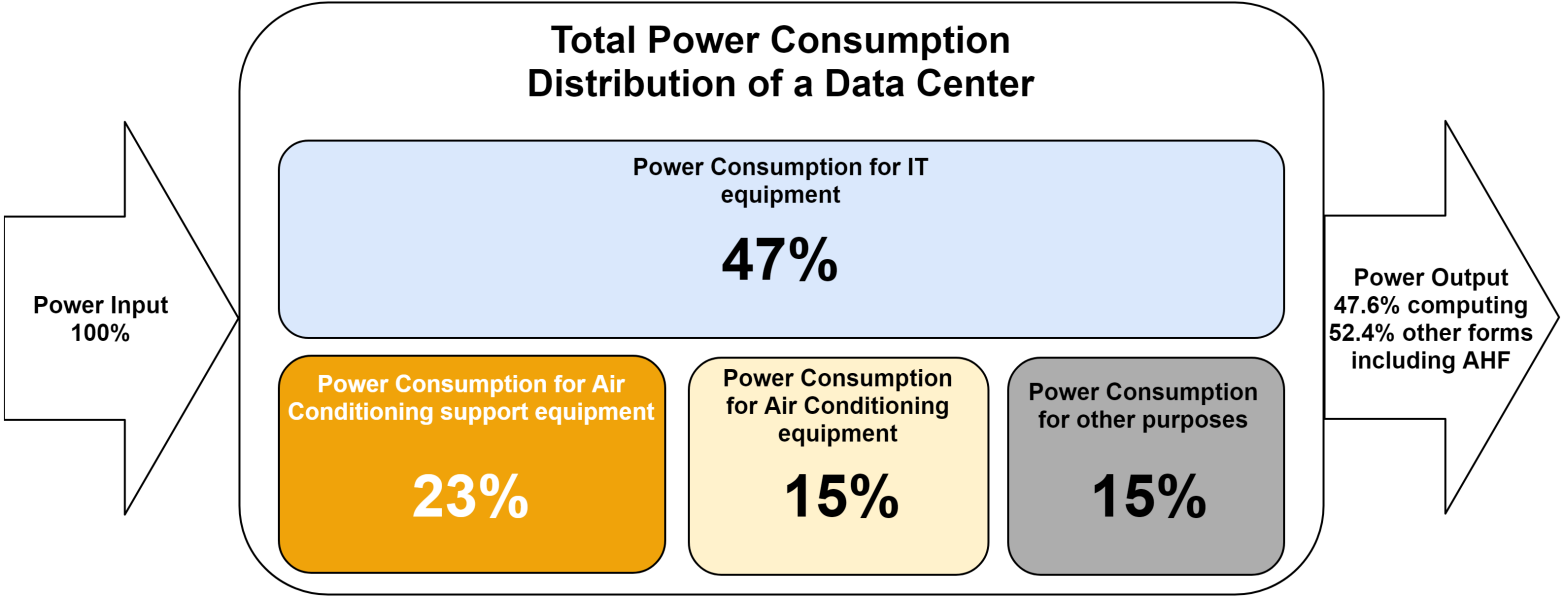
Distribution of electricity consumption in a typical DC with power usage effectiveness (PUE) = 2.1 kW kW^−1^.

The data center industry uses the Power Usage Effectiveness (PUE) metric to measure a DC efficiency (Gadgil & Rahn, 2019). The PUE of a DC is the ratio of the data centre total energy consumption *P*_*Total*_ to information technology equipment energy consumption *P*_*IT*_, calculated, measured or assessed across the same period (ISO/IEC 30134-2:2016, 2018). PUE is calculated using Eq. (1) (Gadgil & Rahn, 2019). The distribution of power consumption in a typical DC was described by Rasmussen (2006), and a schematic representation is depicted in Fig. 3.

(1) \begin{eqnarray*}PUE= \frac{{P}_{Total}}{{P}_{IT}} .\end{eqnarray*}

Two experiments were conducted by Marcinichen, Olivier & Thome (2012) to determine the efficiency of a DC consuming a total power of 900.8 W and 1991 W. The power consumed to perform computational tasks and maintain the IT equipment was found to be 428.1 W and 890 W, respectively. These results indicated a PUE value of ≈ 2.10 and 2.24, respectively. These PUE values are in good agreement with the experiments conducted two years later by Zhao et al. (2014), which resulted in PUE value ≈ 2 for the studied DC. These measures are higher than typical PUE values recently averaged by 1.67 (Ponnusamy, Sharma & Lee, 2021). Additionally, those studies did not use the ISO 30134-2 definition of PUE, which must be measured for one year and where the energy meters are located. Nonetheless, more efficient DCs may waste only around 10% of the consumed energy (Wahlroos et al., 2018) resulting in a lower PUE value, and thus a higher efficiency.

Estimations regarding waste energy in different DCs can fluctuate depending on the fluctuation of workloads, the precise gross floor space (GFS) (Boehme, Berger & Massier, 2015), and the number of CPUs/machines. Thus, public CSPs tend to provide *average* PUE values for all their infrastructure as an indicator of the average efficiency of all their equipment. Google official reports (Google, 2020) claimed an average PUE value of 1.10 during the past 12 months (as of Nov.2020). On the other hand, Microsoft average PUE was reported to be 1.25 (Diekmann, 2020), whereas the most recent report about Amazon cloud PUE indicated an average value of 1.14 (Mah, 2016). The fraction of energy waste (= 1–1/PUE) indicate roughly 9.1%, 20.0%, and 12.3% energy waste by the three CSPs facilities, respectively.

Modeling DCs and their required cooling systems are mathematically presented in details in Uchechukwu, Li & Shen (2014). Specifically, detailed criteria related to cooling technologies and energy consumption of DCs are analyzed in Rasmussen (2005). Generally, the temperature of the air in the servers should be kept in the range of 15–20 °C (Ashrae, 2015). However, the cooling process transfers heat into the surroundings of the center in the form of AHF. Typically, cooling systems require large supply of energy (i.e., electrical, mechanical, etc.), resulting in significant amounts of emitted heat flux.

The heat flux resulting from different types of DC processors was reported in the literature, such as in Patel (2003); Marcinichen, Olivier & Thome (2012); Trutassanawin et al. (2006); Samadiani, Joshi & Mistree (2008). However, the total waste heat generated by those DCs was rarely investigated due to the variety of DCs’ architecture and cooling approaches. Jain et al. (2013), for instance, described how cooling systems in public cloud’s DCs run 365 days a year for full 24 h per day, and how to calculate the waste energy, performance per watt, the greenness of the cloud system, among other criteria of the clouds. They also described major solutions proposed in the literature to decrease the energy consumption of such systems. On the other hand, Huang et al. (2020) analysis revealed that the quality of waste heat in two-phase cooled systems is the highest among the three types of the analyzed cooling systems, due to the higher heat transfer efficiencies. That is, using a two-phase cooling system allows for better waste heat in terms of quality, which leads to higher revenue in case the waste heat was harvested. For air-cooled systems, the quality of waste heat is low, and thus heat pumps, e.g., Bamigbetan et al. (2019), may be added to the system if the waste heat is to be harvested.

### Global AHF estimations

The average global warming is reported to be 1 °C above pre-industrial levels (Delmotte et al., 2018), and is expected to go up in the foreseeable future (Saklani & Khurana, 2019) to reach a minimum of 4 °C above pre-industrial levels by 2100 if the current conditions remain Stott et al. (2006). Several workers around the world investigated this phenomenon and related parameters, including AHF emissions, and proposed potential solutions for the global warming problem. For example, Stewart & Oke (2012) proposed a classification of Local Climate Zones (LCZs) as a model for a comprehensive climate-based classification of urban and rural sites, and suggested 17 different classes of LCZs depending on different parameters, one of which is anthropogenic heat emissions. On a local-scale level, Gabey, Grimmond & Capel-Timms (2019) compared simple and detailed AHF models in London at 500 m^2^ spatial resolution (i.e., they divided the city area into blocks, each with 500 m^2^ area). The authors used population data, national and local energy statistics, and traffic on the roads network data in their analytical study. Accordingly, they measured high levels of AHF in the city center and main roads (roughly 10% of the city area), which accounted for 30–40% of total energy consumption of the city.

On the continental and global scales, Flanner (2009) investigated the AHF over three regions, namely the continental United States, Europe and East Asia, and demonstrated that AHF may indeed contribute to the global warming, and hence should be included in climate simulations. Here, Flanner analyzed the AHF effect in the period of 2005–2100 using historical and real-life measurements. The 2005 results revealed averaged AHF densities of 0.39, 0.68, and 0.22 W m^−2^, respectively, which were predicted to increase in 2040 up to 0.59, 0.89, and 0.76 W m^−2^, respectively. The results of the study also predicted an annual-mean warming of 0.4–0.9 °C over large industrialized regions in 2100 as a result of global AHF emissions. Finally, the author reported Global-mean AHF densities of 0.028, 0.059, and 0.19 W m^−2^ in the years 2005, 2040, and 2100, respectively. Recently, however, global AHF densities in 2020 were reported as 0.12 W m^−2^ (Lu et al., 2017), and 0.15 W m^−2^ (Jin, Wang & Wang, 2020), significantly higher than the value that can be estimated by extrapolating Flanner’s results.

### Solutions for utilizing AHF

Bamigbetan et al. (2019) proposed the employment of a heat pump to transform the waste heat into a usable form. Similarly, Deymi-Dashtebayaz & Valipour-Namanlo (2019) investigated the feasibility of using the air source heat pump (ASHP), as a waste heat recovery system operating with different refrigerants, in a 54-racks data center. The study indicated a consequent 35,000 m^3^/year natural gas saving, 20.8 MWh/year electrical energy saving, 121 tons/year CO_2_ emission reduction, and 25,000$/year cost saving.

Sahana, Bose & Sarddar (2018) proposed positioning a group of sensors into cloud DCs to monitor and minimize cooling efforts, and hence contribute to the energy efficiency of the cloud. Yu et al. (2013) proposed an algorithm to predict the temperature of cloud DCs, in order to facilitate the deployment of energy efficient solutions and predicting energy consumption. Sobhanayak & Turuk (2019) proposed a thermal-aware task allocation and scheduling algorithm for cloud DCs, aiming to minimize power consumption that is involved in cooling processes.

Pärssinen et al. (2019) provided an economic investment assessment of DC waste heat utilization in three different size cases, namely small, medium and large. Accordingly, they concluded that medium and large cases (e.g., DCs of public CSPs) can be considered profitable business opportunities and should result in a decision to invest in a waste heat reuse solution. For the biggest barriers for utilizing waste heat are the low quality of waste heat and high investment costs, Wahlroos et al. (2018) proposed A systematic 8-step change process to ensure success in changing the priority of AHF utilization in the DC and district heating market. Similarly, Alhamwi et al. (2018) showed how investing in local electricity storage and on-site renewable power generation solutions can significantly reduce the total DC system costs.

## Methods

In our analysis, we use 933 DCs distributed over the three CSPs in accordance with the data in Table 1, and adopt the energy consumption per DC based on Kaplan’s estimate (≈101.7 × 10^6^ kWh/year). Assuming that all energy forms not used for IT purposes are transformed into heat, the PUE values reported for the three CSPs (‘Power distribution and efficiency in public data centers’) were used to determine the fraction of the energy wasted (out of total consumed) using Eq. (1). According to the regional Geo-distribution of the studied CSPs, the continental and global shares of waste heat, contributed by each CSP, were evaluated and listed in Table 3. Subsequently, we compute the cumulative continental AHF densities per square meter using Eq. (2), where *w* is the waste heat (MW), *S* is the corresponding area of the continent (*m*^2^) and *c* is the ratio of waste energy gained by Earth, at the Earth’s surface and close-to-surface atmosphere, to the total received energy. To obtain accurate and individual values of *c*, we used the monthly Surface Net Solar Radiation (SNSR) data with a spatial resolution of 7.5 arc-minutes, reported in the ERA-Interim reanalysis dataset of the European Centre for Medium-Range Weather Forecasts (http://apps.ecmwf.int/datasets/). SNSR values represent the energy available to/gained by Earth, at the Earth’s surface and close-to-surface atmosphere layer, out of the total received solar energy (Verma et al., 2016). That is, we divide cumulative continental waste heat by the area of the corresponding continent (McColl, 2014), and we sum up those shares to deduce total continental AHF densities contributed by all of the three studied CSPs.^4^ 4Since there were no DCs located in Eastern European countries (such as Russia), we excluded its area from our computations.Table 4 presents detailed data obtained by these calculations, in addition to AHF densities originated by all Anthropogenic activities according to a recent report introduced in Jin, Wang & Wang (2020).

(2) \begin{eqnarray*}AHF= \frac{w\times c}{S} \end{eqnarray*}

**Table 3:** Continental and global rates of waste heat contributed by the top three cloud service providers.

CSP	**Continent**	**No.Regions**	**No.DCs**	***P*_*Total*_** (MW)	**PUE**	**Waste Heat** (MW)
	N. America	7	63	730.8		89.8884
	S. America	1	9	104.4		12.8412
	Asia	8	66	765.6	**1.14**	94.1688
**Amazon**	Europe	6	54	626.4		77.0472
	Oceania	1	9	104.4		12.8412
	Africa	1	9	104.4		12.8412
	**Total**	**26**	**210**	** 2,436**	–	**299.628**
	N. America	18	162	1,879.2		375.84
	S. America	1	12	139.2		27.84
	Asia	17	153	1,774.8	**1.25**	354.96
**M.Azure**	Europe	15	135	1,566		313.2
	Oceania	4	40	464		92.8
	Africa	2	20	232		46.4
	**Total**	**57**	**522**	**6,055.2**	–	**1,211.04**
	N. America	8	70	812		73.08
	S. America	1	9	104.4		9.396
**Google**	Asia	7	60	696	**1.10**	62.64
	Europe	6	53	614.8		55.332
	Oceania	1	9	104.4		9.396
	**Total**	**23**	**201**	**2,331.6**	–	**209.844**
Global		**106**	**933**	**10,822.8**	–	**1,720.512**

## Results and Discussion

### Current AHF emissions by public CSPs

**Figure 4:**
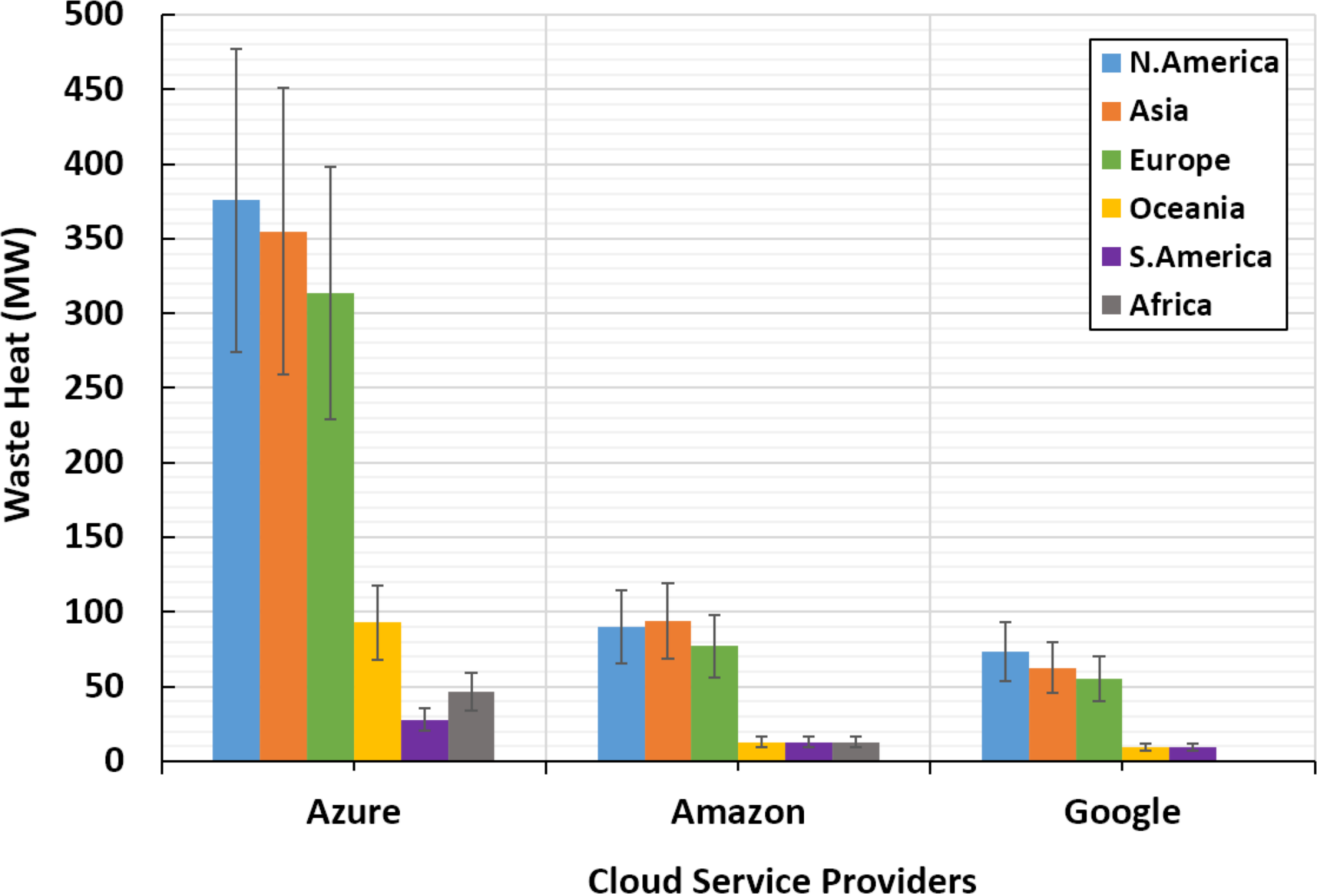
Detailed waste heat amounts of top cloud service providers in different continents.

**Figure 5:**
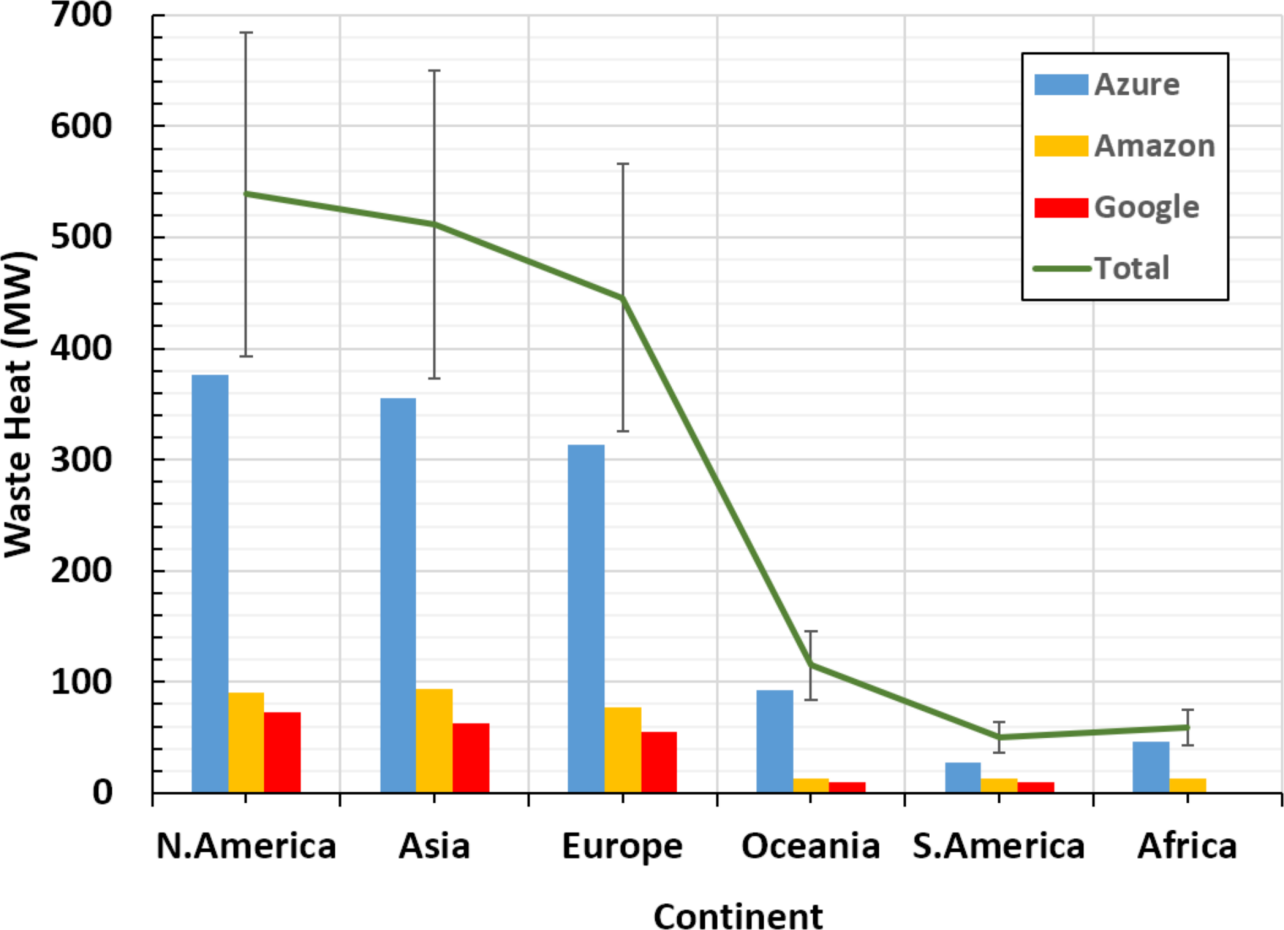
Continental waste heat emissions by top cloud service providers.

**Figure 6:**
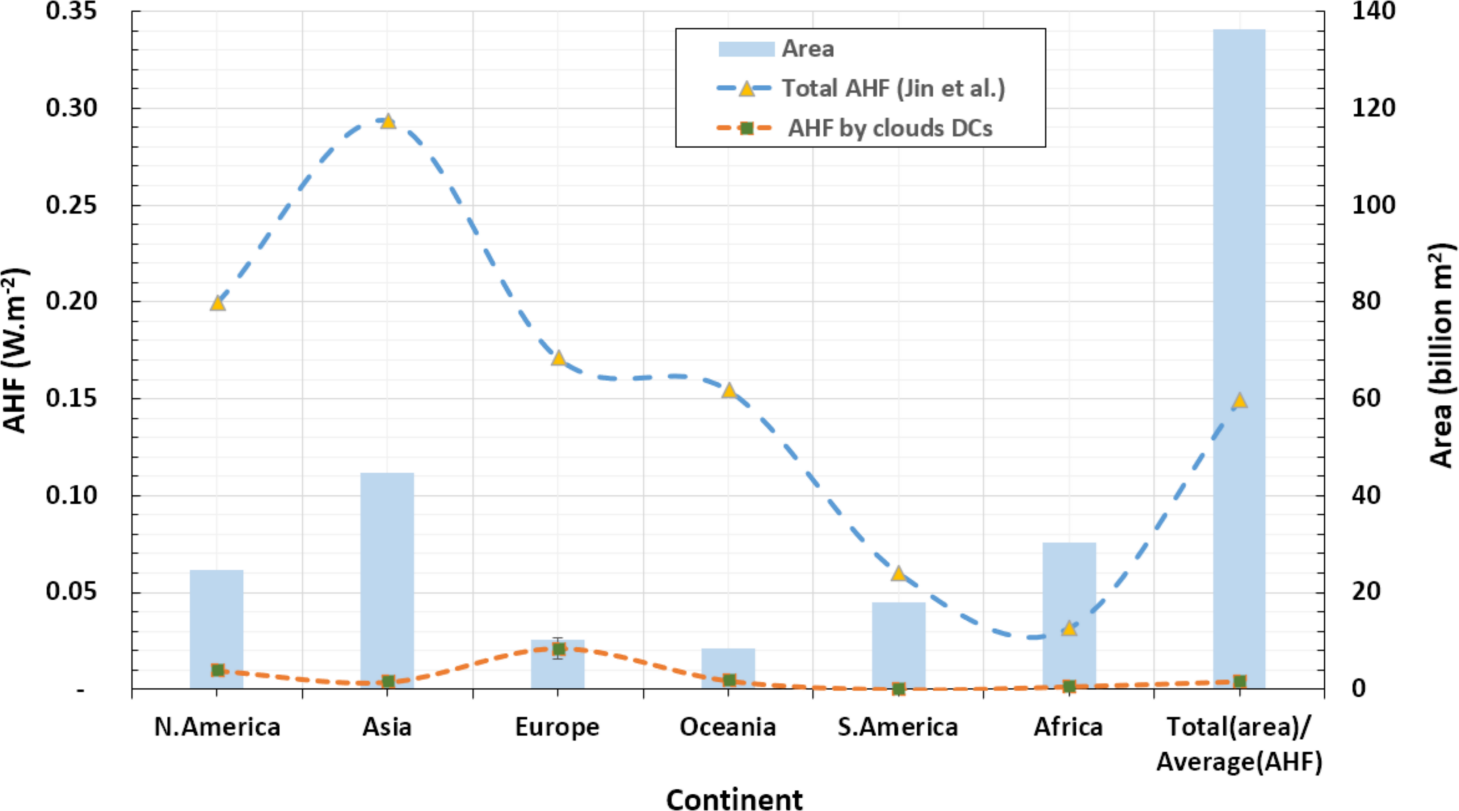
Mean continental AHF density emitted by top public cloud service providers against average total AHF emissions estimated by Jin, Wang & Wang (2020) (Wm^−2^: represented by curves correlated with the primary *y*-axis on the left). Area values of different continents (Billion m^2^: represented by blue bars correlated with the secondary *y*-axis on the right).

The results in Tables 3 and 4 indicate that:

1. With a total waste heat at a rate of 1,211.04 MW, Microsoft Azure contributes more than Amazon and Google collectively, whose contributions are 299.628 and 209.844 MW, respectively. The contributions of the individual CSPs relative to the cumulative waste heat by the three CSPs are, respectively, 70.4%, 17.4% and 12.2%. Interestingly, these values could not be expected according to the market shares discussed in ‘Power consumption of public cloud data centers’.
2. Azure and Google’s highest waste heat rates are in the continents of N.America, Asia, and Europe, respectively. While the highest waste heat rates emitted by Amazon are in Asia, N.America, and Europe, respectively. Figure 4 illustrates these results.
3. An average of 2.88% out of total continental AHF density is contributed by DCs that belong to the studied CSPs (i.e., Azure, Amazon and Google). This percentage can be divided using the ratios presented in point 1 above.
4. Although Europe receives less total waste heat than N.America and Asia, as illustrated in Fig. 5, it receives the highest AHF density due to its relatively small area compared to N.America and Asia. That is, Europe atmosphere receives more than 25% out of the total waste heat emissions by all DCs under consideration. On the other hand, lowest AHF densities emitted by public DCs can be found in S.America, Asia and Africa, respectively. This is due to their large areas that include small number of public DCs, and/or low SNSR values. These results are illustrated in Fig. 6.

### Future trends of AHF emissions of public clouds DCs

**Figure 7:**
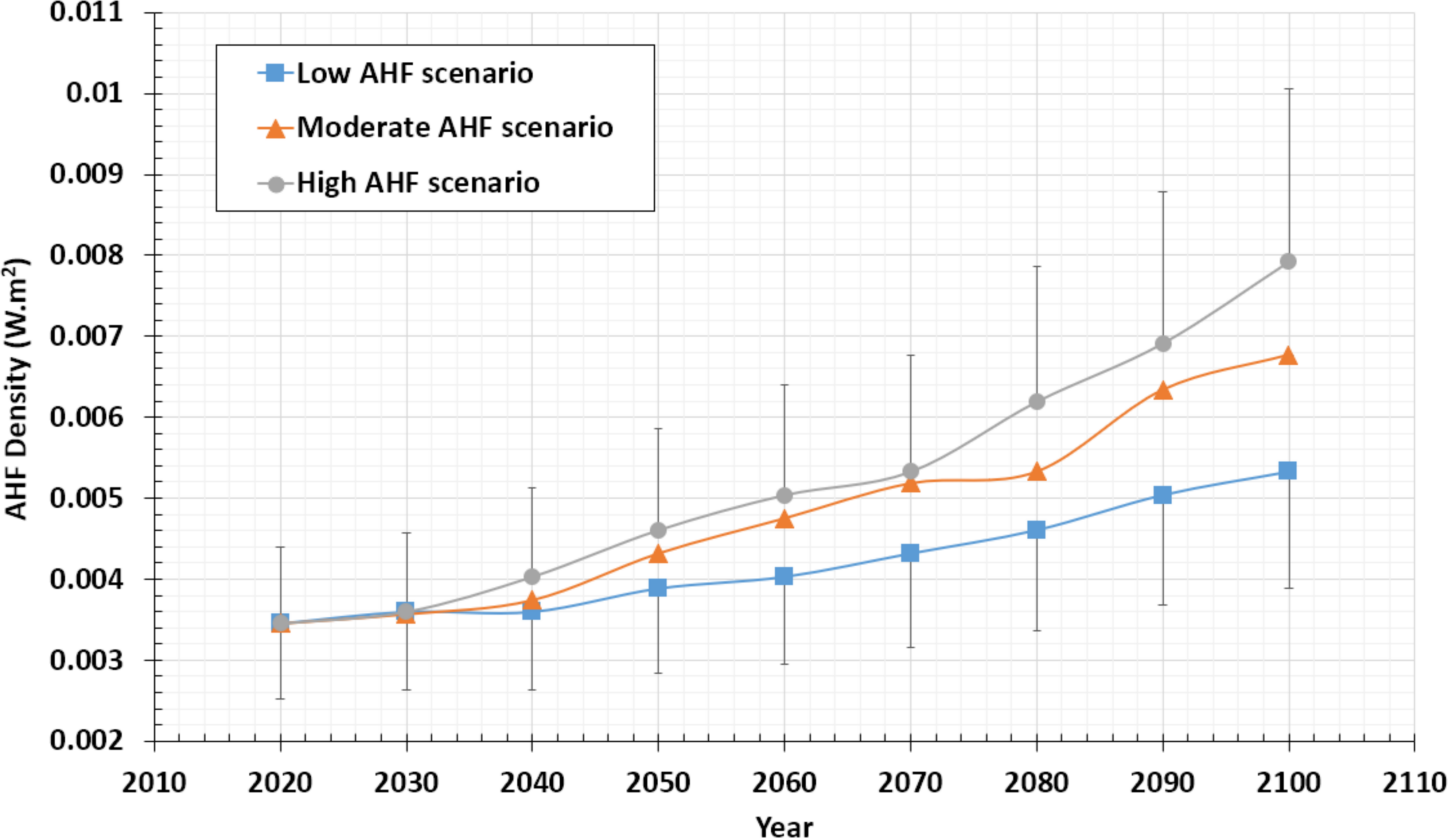
Future trends of global AHF emissions by public cloud DCs in the period [2020–2100]. Values estimated by taking 2.88% (± 27%) out of global AHF future trends estimated by Lu et al. (2017).

Based on a top-down energy inventory approach and up-to-date data, Lu et al. (2017) provided globally averaged AHF estimations up until the year 2100, considering high, moderate, and low AHF emissions scenarios. On the other hand, the percentage of averaged current continental AHF, emitted by the studied CSPs, relative to averaged current continental AHF of all activities, was calculated in Table 4 and equals 2.88%. Consequently, we deploy this percentage into Lu et al.’s estimations, to deduce future trends of AHF of, specifically, DCs of the studied public CSPs. Similarly, Fig. 7 demonstrates our estimations concerning high, moderate, and low growth in the Data Center Industry, until the year 2100.

**Table 4:** Continental AHF density emitted by top public cloud DCs against average total AHF emissions proposed by Jin, Wang & Wang (2020).

**Continent**	**Cumulative** Waste Heat (MW)	**Area** (Billion m^2^)	**SNSR**	**AHF Density by** public DCs (W/m^2^)	**Total AHF Density** (W/m^2^) (Jin, Wang & Wang, 2020)	*Pct.*
N.America	538.8084	24.71	0.44	0.00959	0.19	5.05%
S.America	50.0772	17.84	−0.01	−0.00003	0.06	−0.05%
Asia	511.7688	44.58	−0.33	−0.00379	0.29	−1.31%
Europe	445.5792	10.18	0.48	0.02101	0.15	14.01%
Oceania	115.0372	8.5	0.33	0.00447	0.15	2.98%
Africa	59.2412	30.37	0.78	0.00152	0.03	5.07%
**Total**/ **Average:**	**1,720.512**	**136.18**	**0.33**	**0.00417**	**0.145**	**2.88%**

As can be noted in the figure, average continental AHF, emitted by public clouds DCs is expected to reach a value between 0.005 and 0.008 (± 27%) Wm^−2^. Such AHF value represents a percentage of 2.63–4.21% of the mean global AHF density estimated by Flanner.

## Conclusion

In this work, we have investigated Anthropogenic Heat Flux (AHF) of top Cloud Service Providers (CSP) (i.e., Microsoft Azure, Google, and Amazon), caused by each CSP’s DCs. We have collected published data about energy consumed by public DCs, and projected the placement of CSPs regions on a global heat flux map. Subsequently, we computed the continental and global AHF caused by all CSPs using the Power Usage Effectiveness (PUE) index and the Surface Net Solar Radiation (SNSR). Our investigation led to key observations about AHF emissions of top CSPs’ DCs. We found that these CSPs waste a total energy, in the form of heat, at a rate of 1,720.512 MW. Accordingly, we found that 2.88% of average global AHF is caused by the concerned CSPs. Additionally, we found that Europe’s atmosphere receives the highest amount of AHF density per square meter, although North America’s atmosphere receives the absolute highest amount of waste heat.

According to our findings, AHF was found to be an active influential factor on continental and regional climate. Flanner predictions Flanner (2009) strongly suggested taking AHF into account when simulating and computing climate changes. In 2005, the Kyoto Protocol Grubb et al. (1999) was enforced, in a potential to govern GHG emissions, by allowing each country to use a limited quota for their industries. Such limitations were accepted and adopted by many countries around the world, for encouraging the control over the global warming problem. Similarly, international agreements regarding AHF quota per industry, or per country, can be enforced to further approach the control of the Global Warming problem. For example, if some CSPs are partly using renewable energy, this will reduce the greenhouse effect, since part of this energy is consumed by the DCs operations rather than contributing to greenhouse effects. That is, reducing the natural heat effect, while increasing the AHF, may lead to Zero net change. In optimistic scenarios, such climate-aware practices may indeed reduce the averaged total AHF values.

We do recommend including AHF quotas in future adjustments to the Kyoto Protocol, or any similar—global warming concerned—international consortium. Nevertheless, our present research only sheds light on a fractional source of AHF. We found that, on average, 2.88% of average continental AHF is emitted by the top three CSPs. We encourage researchers to conduct wider studies for determining required parameters to facilitate the inclusion of AHF considerations in future international protocols. Such studies may include defining Regional and Continental Tolerable Capacities, by performing quantitative and qualitative analysis of regional and continental local atmospheres and by referring to recognizable standard classification systems, such as the LCZ system. For example, members of the European Union may decide to keep the LCZ classification of Europe within the range of LCZ-1 class or lower. Such decision may be made according to specific criteria, such as the Human Thermal Comfort (HTC) preferences (Gal & Kntor, 2020). Accordingly, stronger measures and laws need to be enforced if it was found that the total AHF emissions, by different sectors, is being pushed towards this class.

Forcing international quotas does not necessarily mean limiting the operations of AHF sources, as much as deploying efficient mechanisms to comply with the enforced international agreements/quotas. On the other hand, adopting modern technologies to harvest and convert AHF into high quality/usable power (some of which were mentioned in ‘Background’) was proven to be profitable in the case of public clouds. With all the results we obtained in this work, we are motivated to use similar methodologies in the future, to assess AHF emitted by other sources, such as power plants and industrial factories.

## Additional Information and Declarations

### Data Availability

The following information was supplied regarding data availability: The collated data are available in Tables 1–4.
